# Estrogen Induces Global Reorganization of Chromatin Structure in Human Breast Cancer Cells

**DOI:** 10.1371/journal.pone.0113354

**Published:** 2014-12-03

**Authors:** Raphaël Mourad, Pei-Yin Hsu, Liran Juan, Changyu Shen, Prasad Koneru, Hai Lin, Yunlong Liu, Kenneth Nephew, Tim H. Huang, Lang Li

**Affiliations:** 1 Department of Medical and Molecular Genetics, Center for Computational Biology and Bioinformatics, Indiana School of Medicine, Indiana University, Indianapolis, IN, 46202, United States of America; 2 Department of Biostatistics, Center for Computational Biology and Bioinformatics, Indiana School of Medicine, Indiana University, Indianapolis, IN, 46202, United States of America; 3 Departments of Molecular Medicine/Institute of Biotechnology, University of Texas Health Science Center, San Antonio, TX, 78245, United States of America; 4 Laboratory of Ovarian Cancer Epigenomics, Indiana University, Bloomington, IN, 47405, United States of America; University of Wisconsin - Madison, United States of America

## Abstract

In the cell nucleus, each chromosome is confined to a chromosome territory. This spatial organization of chromosomes plays a crucial role in gene regulation and genome stability. An additional level of organization has been discovered at the chromosome scale: the spatial segregation into open and closed chromatins to form two genome-wide compartments. Although considerable progress has been made in our knowledge of chromatin organization, a fundamental issue remains the understanding of its dynamics, especially in cancer. To address this issue, we performed genome-wide mapping of chromatin interactions (Hi-C) over the time after estrogen stimulation of breast cancer cells. To biologically interpret these interactions, we integrated with estrogen receptor 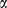 (ER*α*) binding events, gene expression and epigenetic marks. We show that gene-rich chromosomes as well as areas of open and highly transcribed chromatins are rearranged to greater spatial proximity, thus enabling genes to share transcriptional machinery and regulatory elements. At a smaller scale, differentially interacting loci are enriched for cancer proliferation and estrogen-related genes. Moreover, these loci are correlated with higher ER*α* binding events and gene expression. Taken together these results reveal the role of a hormone - estrogen - on genome organization, and its effect on gene regulation in cancer.

## Introduction

Each chromosome is confined to a specific chromosome territory (CT) in the cell nucleus. This spatial organization of genome plays a crucial role in gene regulation and genome stability [1]–[5]. Using high-throughput chromosome conformation capture (Hi-C), Lieberman *et al.* confirmed the presence of CTs in human genome but also revealed that, at the chromosome scale, the genome organization is characterized by the spatial segregation of open and closed chromatins to form two genome-wide compartments (named A and B) [6], [7]. Contrary to the second compartment (B), the first compartment (A) is associated with the presence of genes, high expression and accessible chromatin. Moreover the first compartment is correlated with both activating and repressive chromatin marks. Similar chromatin organization was observed in the *Drosophila* genome [8]. At the megabase scale, chromatin organization is consistent with a fractal globule polymer model [7]. The fractal globule polymer model is attractive as it enables maximally dense packing while preserving the ability to easily fold and unfold any genomic locus, an essential feature in gene expression regulation and cell cycle [9], [10]. Using a deeper sequencing than Lieberman *et al.*, Dixon *et al.* identified topologically associating domains (TADs) showing the existence of highly self-interacting regions bounded by narrow segments [5], [11]. These TADs represent a pervasive structural feature of the genome organization. The domains are stable across different cell types and highly conserved across species. The integration of Hi-C data with numerous types of data (DNase-seq, ChIP-seq for transcription factors and histone modifications) showed that interacting loci can be classified in 12 different profiles [12]. Moreover the high correlation of Hi-C data with the binding of CCCTC-binding factor (CTCF) to the chromatin suggests that CTCF is a major organizer of both the structure of chromosomal fiber within each individual chromosome and of chromosome territories within the cell nucleus [13].

Hi-C is a recent high-throughput chromosome conformation capture technology for studying genome folding [7], [14]. Hi-C improves the previous technologies 3C (chromosome conformation capture) [15], Circular Chromosome Conformation capture (4C) [6], [16] and Chromosome Conformation Capture Carbon Copy (5C) [17] by allowing unbiased genome-wide analysis of chromatin interactions. More recently, Tethered Conformation Capture (TCC) has been developed to improve signal-to-noise ratio by performing ligations on solid substrates rather than in solution [18]. As an alternative approach to Hi-C and TCC, the Chromatin Interaction Analysis by Paired-End Tag Sequencing (ChIA-PET) combines 3C with immunoprecipitation and is thus more suitable for the analysis of functional chromatin interaction networks [19], [20].

The analysis of Hi-C data is complex, and many statistical and computational methods have been recently developed to correct interaction heatmaps for several biases such as GC-content and distance between reads [21]–[24], to identify chromatin compartments [7], [22], to visualize Hi-C networks [25] and to 3D model chromosome folding [7], [8], [26], [27].

Although considerable progress has been made in our knowledge of global chromatin organization, a fundamental issue remains the understanding of its dynamics. There is a growing body of evidence that changes in transcriptional activity of genes is associated with repositioning of chromosomal regions relative to nuclear compartments and other genomic loci [2], [28], [29]. Moreover, several contacts between different chromosomal loci have been documented, a phenomenon called chromosome kissing [30]. Conversely, it has been shown that global chromosome positions are transmitted through mitosis in mammalian cells [31]. Another related issue is whether a molecule such as a hormone can stimulate the dynamics of chromatin organization, since we know that hormones have strong effects on gene activity. Current approaches to address these questions have involved fluorescence microscopy such as FISH but present the drawback not to provide a high resolution as Hi-C does.

To answer these issues, we selected a breast cancer cell, MCF-7, and utilized Hi-C technology to capture chromatin organization before and after 17*β*-estradiol (E2) treatment. MCF-7 cell is an established cell system to investigate genome-wide estrogen mediated signaling pathways [32], their associated histone modification mechanisms [33] and DNA methylation landscape [34]. We previously reported gene expression regulation through DNA looping after E2 stimulation [35], [36], suggesting that chromatin interaction is a viable epigenetic mechanism of MCF7 cell response to E2 stimulation. Moreover it has recently been shown that for estrogen-repressed genes, estrogen treatment leads to chromatin structure reconfiguration, thereby disrupting the originally transcription-efficient chromatin structures [37]. Besides, from a more global point of view, E2 is known to alter the large-scale chromatin structure [38]. In other cancer cells - normal benign prostate epithelial cell lines (RWPE1) - it has been demonstrated that an oncogenic transcription factor (ERG) can induce changes in chromatin organization [39].

In this study, we show that time-series Hi-C data analysis is a promising methodology for better understanding global dynamics of chromatin and its link with gene regulation. Beside augmenting the number of long-range interactions, E2 induces a dynamic mechanism of global chromatin reorganization. To interpret this global chromatin reorganization, we compare Hi-C data with ER*α* binding, gene expression and multiple epigenetic marks. More specifically, gene-rich chromosomes as well as areas of open and highly transcribed chromatins are rearranged to greater spatial proximity. This phenomenon then allows genes to share transcriptional machinery and regulatory elements. At a smaller scale, loci that are differentially interacting show enrichment for cancer proliferation and E2-related genes. In addition, these loci are involved with higher ER*α* binding events and gene expression. Based on these results, our study demonstrates the role of a hormone - estrogen - on global genome organization and its link with gene regulantion in cancer.

## Materials and Methods

### Hi-C experiment

An ER-positive breast cancer cell line - MCF-7 - was obtained from the American Type Culture Collection and maintained in phenol red-free medium. After 48 hours of hormone deprivation in 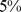 charcoal-dextran stripped serum media (no phenol red), cells were stimulated with 70 nM estrogen (E2) in different time periods (0, 0.5, 1, 4, 24 hours). To check for synchronization of cells, we assessed expression of *TFF1* using RT-qPCR data in three different batches of cells (Figure S1 in File S1).

For each time point, there are two biological replicate samples and two lanes per sample (*i.e.* 4 lanes per time point). Each biological replicate was then subjected to genome-wide chromosome conformation capture (Hi-C) as previously described [7]. Briefly, cells were fixed with 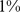 formaldehyde. Chromatin was digested with HindIII (NEB, Ipswich, MA). DNA ends of digested chromatin were marked by biotin-14-dCTP (Invitrogen, Carlsbad, CA) following blunt-end ligation with T4 DNA ligase in diluted condition. Ligated DNA was then de-crosslinked and purified by phenol extraction procedures. Biotin-14-dCTP at non-ligated DNA ends was removed with exonuclease activity of T4 DNA polymerase. Ligated DNA was then applied to paired-end sequencing by using the Illumina sequencing technology platform (Genome Analyzer IIx, Illumina).

Sample preparation for paired-end sequencing was performed following the manufacturer's instructions. Briefly, ligated DNA (5* µ*g) was sheared to a size of 300–500 basepairs by a nebulizer supplied with the Illumina paired-end sample preparation kit. Fragmented DNA was end-paired using T4 DNA polymerase and Klenow polymerase with T4 polynucleotide kinase to phosphorylate the 5′ ends. A 3′ overhang was created using a 3′-5′ exonuclease-deficient Klenow fragment, and then subjected to immunoprecipitation by Dynabeads MyOne Streptavin C1 Beads (Invitrogen) in DNA LoBind tubes (Eppendorf, Westbury, NY) with ligation of Illumina paired-end adaptor oligonucleotides. The ligation mixtures were electrophoresed on E-gel SizeSelect 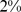 pre-cast agarose gels (Invitrogen) to collect 250-bp fragments. Size-selected DNA fragments were enriched with Illumina paired-end primers by a 15-cycle PCR reaction. DNA samples (20 nM per sample), quantified by an Agilent Bioanalyzer, were loaded onto the paired-end flowcell of Genome Analyzer IIx (GAIIx) in the supplied cluster station according to the manufacturer's protocol. Clusters of PCR colonies were then sequenced on GAIIx with 51-bp per read.

### Hi-C data preprocessing

From the Hi-C experiment, reads were preprocessed in five steps to calculate the interaction matrices. Steps (i), (ii) and (iv) were done using Mirny's lab library [22] following their standard protocol (http://mirnylab.bitbucket.org/hiclib/index.html). The five preprocessing steps are the following: (i) the raw sequences of both ends of Hi-C molecules are first mapped separately to the human genome reference hg19 using the Bowtie2 aligning software; (ii) after alignment, the Mirny's lab pipeline discards read pairs with two unmapped sides and removes read pairs corresponding to repeated instances of the same DNA molecule (which may result from PCR amplification). The pipeline then analyzes the position and direction of each mappable read from each read pair to separate molecular byproducts from informative double-sided (DS) reads; (iii) finally, all translocated and amplified regions [40] are removed from the Hi-C data in order to deal with structural variations. Next the number 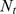 of short-range interactions (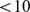 kb), as well as the number of long-range interactions (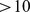 kb), are calculated for each time point 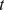; (iv) then reads of the different lanes at a specific time point are merged and filtered at the bin level, for different resolutions (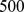 kb, 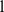 Mb, 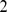 Mb and 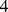 Mb). DS reads are assigned to a genome-versus-genome heatmap and single-sided (SS) reads to a genome-wide vector. Iterative correction is next done on data for each time point to remove all types of biases; (v) interaction matrices at time 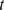 are normalized by a factor 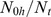.

The number of reads obtained after Hi-C data preprocessing is summarized in Table S1a in File S1 (lane level), Table S1b in File S1 (replicate level) and Table S1c in File S1 (time point level). The Tables S1a and S1b in File S1 show that interaction counts are similar across lanes and across replicates, respectively. High correlations between replicate heatmaps were observed (r = 0.83, r = 0.93, r = 0.94, r = 0.95, r = 0.92, for 0 h, 0.5 h, 1 h, 4 h and 24 h, respectively; correlations were calculated for intrachromosomal interactions only). After all filterings, there were 13835097, 15759919, 13222156, 14763310 and 11193041 interactions for the time points 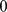, 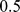, 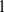, 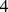 and 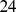 hours, respectively.

All Hi-C data are available at GEO (http://www.ncbi.nlm.nih.gov/geo/) using accession number GSE51687.

### Genetic and epigenetic information

For H3K4Me2 binding data, we used ChIP-seq results from MCF-7 cells which were not stimulated with E2 [41]. We also downloaded several tracks from UCSC genome browser (http://genome.ucsc.edu/): DNaseI hypersensitivity [42], [43], CTCF binding sites [42], DNA methylation [44] and gene location. All these data are from MCF-7 cells which were not stimulated with E2, since the data are mostly static in response to E2 after only 24 h [45]–[47]. For gene expression, we used time-series MCF-7 data after E2 stimulation from [48]. Time points 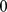, 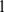, 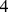 and 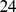 h are shared between Hi-C and gene expression data. For RNA polymerase II binding, we analyzed ChIP-seq data at time points 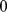 and 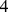 h from [49]. For estrogen receptor 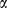 ChIP-seq, we used time series MCF-7 data after E2 stimulation [50]. Shared time points with Hi-C data are 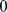 and 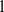 h. All data were mapped to human genome reference hg19.

### Shannon entropy calculation

Shannon entropy is calculated as:
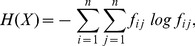
(1)where 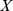 is the upper (or lower) triangular matrix of interaction frequency (normalized by dividing the matrix by the sum of all the matrix cells), 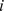 and 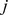 represents the row and column indices of X, and 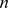 the number of rows (or colums) of the matrix 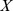. Each 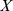 cell value 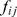 is the interaction frequency between the two bins 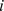 and 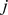.

The interpretation of entropy is very simple. In statistics, it is used to evaluate the dispersion of a distribution. For Hi-C data analysis, the higher the entropy the higher is the spatial dispersion of interactions.

### Chromosome territories

Chromosomes which are close to each other are likely to interact more. To evaluate proximity, we consider the normalized frequency of interactions between two chromosomes 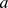 and 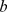:
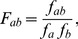
(2)with 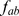 the frequency of interactions between the two chromosomes 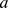 and 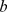, 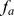 and 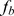 the marginal frequencies of interactions for the chromosomes 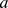 and 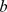, respectively. For a chromosome, the marginal frequency is the sum of frequencies of interactions between the chromosome and all the other chromosomes.

### Scaling coefficient estimation

To estimate the scaling coefficient, we first calculate the genome-wide average intrachromosomal interaction frequency 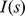 for each distance 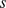. Then, for the interval 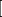
500 kb, 7 Mb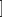, the log-log regression between 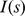 and 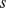 is calculated. From the log-log regression, the scaling coefficient is extracted. The calculation is carried out for each time point independently and with a 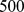 kb binning.

### 3D chromosome modeling

For 3D chromosome modeling, we use the recent program BACH [27] (http://www.people.fas.harvard.edu/~junliu/BACH/). The program uses a Bayesian probabilistic approach which assumes that local genomic region (i.e., a topological domain) of interest exhibits a consensus 3D chromosomal structure in a cell population. BACH relies on an efficient Markov chain Monte Carlo (MCMC) method to infer the underlying consensus 3D chromosomal structure.

### Compartment inference using PCA

We use principal component analysis to infer chromatin compartments [7]. First the matrix of intrachromosomal interactions is used to compute the matrix of correlations. A resolution of 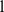 Mb provides enough power for estimating correlations. We calculate Pearson correlation for each pair of rows (or columns) of the matrix of intrachromosomal interactions. Then principal component analysis is applied to this correlation matrix. The first principal component (PC1) reveals the compartmentalization of chromatin. Positions with positive PC1 values belong to the first compartment while positions with negative PC1 values belong to the second compartment. To ensure that, over the time points, the PC1 axis is not flipped (positive values being negative values and *vice versa*), PC1 is constrained to be positively correlated with GC-content.

To calculate correlations between chromatin compartmentalization (principal components) and genetic and epigenetic tracks, we proceed in the following way. We first bin at 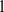 Mb scale the track values. Then, in order to remove the confounding effect of gene density when calculating the correlation with a track, we regress out the gene counts before calculating correlations. We next calculate the Pearson correlation between the principal component and the track.

### Gene annotation and functional enrichment

To detect differentially interacting loci, we use the Wilcoxon test for paired samples to compare each interaction correlation matrix row between the time point t (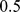, 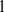, 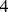 or 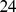 h) and the time point 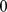 h. Within the loci, active genes were mapped (using UCSC genome browser gene positions, and H3K4Me2 and DNaseI HS marks). Then DAVID program (http://david.abcc.ncifcrf.gov/) determines functional enrichment clusters of gene ontology terms.

Estrogen receptor alpha (ER*α*) binding events are mapped within a window of 250 kb around gene transcription start sites. Enrichment is assessed using an exact binomial test comparing counts of differentially versus non-differentially interacting genes which are bound by ER*α*. ER*α* ChIA-PET interaction events are mapped in the same manner and exact binomial test is used to compare counts of differentially versus non-differentially interacting genes which are ER*α* bound and are reported interacting with ChIA-PET. Microarray data are used to compare expression of differentially versus non-differentially interacting genes using Student's t-test. RNA polymerase II binding events are mapped within a window of 10 kb around gene transcription start sites. Enrichment is assessed using an exact binomial test comparing counts of differentially versus non-differentially interacting genes which are occupied by RNA polymerase II.

### Interacting loci network

To visualize networks of interacting loci, we used the CytoHiC plug-in of Cytoscape (http://www.cl.cam.ac.uk/~ys388/CytoHiC/) [25]. Interacting loci were annotated using gene positions and ER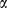 binding sites to visualize networks of interacting genes and networks of ER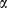 bound interactions, respectively. Network centralization coefficient is computed using Cytoscape.

### Expression analysis of estrogen-responsive genes

After 48 hours of hormone deprivation in 5% charcoal-dextran stripped serum media without phenol red, MCF-7 cells were stimulated with either 10 nM or 70 nM estrogen (E2) in different time periods (0, 0.5, 1, 4, 24 hours) and then subjected to expression analysis by reverse transcription-quantitative PCR (RT-qPCR). Total RNA (2* µ*g) was reversely transcribed to cDNA with oligo-dT (SuperScript III; Invitrogen). RT-qPCR was performed by using SYBR Green dye chemistry (Applied Biosystems) on a StepOnePlus Real-Time PCR System apparatus (Applied Biosystems). Gene expression was measured by the 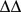
Ct method using *β*-actin as the internal control. Expression for the three genes *CTSD*, *GREB1* and *TFF1* are presented in Figure S2 in File S1. We observed that 70 nM of E2 stimulation has similar effect as 10 nM does (dosage most commonly used).

### Chromatin immunoprecipitation-quantitative PCR (ChIP-qPCR)

After 48 hours of hormone deprivation in 5% charcoal-dextran stripped serum media without phenol red, MCF-7 cells were stimulated with either 10 nM or 70 nM estrogen (E2) in different time periods (0, 0.5, 1, 4, 24 hours) and then subjected to chromatin immunoprecipitation analysis. Immunoprecipitated DNA from treated MCF-7 cells was prepared according to the ChIP protocol published by Young and coworkers [51]. Briefly, treated cells were fixed with 1% formaldehyde at room temperature for 10 min. The resultant DNA-protein complexes were sheared with a Bioruptor (Diagenode, Sparta, NJ) to an average of 450 bp as verified on a 1.5% agarose gel, followed by immunoprecipitation using the Dynabeads Protein G (100.04D; Invitrogen) coated with antibodies specific for ER*α* (Santa Cruz). Pull-down DNA was subjected to quantitative PCR analysis using the SYBR Green-based detection method on a StepOnePlus Real-Time PCR System apparatus (Applied Biosystems). Quantitative values measured by a standard curve (50 to 0.08 ng, 5-fold dilution, R2>0.99) of input DNA amplified with the same primer set. Results are presented as the mean of triplicates with standard derivation. ER*α* binding for the three estrogen-responsive genes *CTSD*, *GREB1* and *TFF1*, and for one control gene *GAPDH*, are presented in Figure S3 in File S1. We observed that 70 nM of E2 stimulation has similar effect as 10 nM does (dosage most commonly used).

### Chromosome conformation capture-quantitative PCR (3C-qPCR)

Charcoal-stripped MCF-7 cells stimulated with E2 (70 nM) were collected at different time-points of treatment (0, 0.5, 1, 4, and 24 hours). Treated cells were then subjected to 3C-qPCR analyses as previously described [52]. Briefly, fixed chromatin by 1% formaldehyde was digested using HindIII, and then ligated by T4 DNA ligase in a diluted condition. Ligated DNA was then de-crosslinked and purified by classical phenol extraction procedures. Real-time PCR was performed on a StepOnePlus Real-Time PCR System apparatus (Applied Biosystems) using the TaqMan technology (QuantiTect Probe PCR Master Mix, Qiagen). We used a 5′FAM-3′BHQ1 oligonucleotidic probe (Invitrogen). To rule out the possibility of false-negative looping occurrence caused by unsuccessful 3C assay, we pooled two human bacterial artificial clones (BAC), mapping the interested regions as the positive control of the 3C-qPCR assays. These BACs were also used to examine the primer efficiency. For data analysis, the Ct obtained for each chimerical ligation fragment was processed using parameters of a standard curve (slope and intercept) from BAC to obtain quantification values that were normalized to a *GAPDH* loading control.

### Interphase Fluorescence In Situ Hybridization (FISH)

E2-treated (70 nM) MCF-7 cells were fixed by Carnoy's fixative and then subjected to Interphase Fluorescence In Situ hybridization (FISH). The probe mapped to *THRAP1* (or *MED13*) and the associated interacting ER*α* binding site localized at 20q13 (20q13 DERE) were prepared from BACs (Invitrogen; RP11-561K8 for *THRAP1* and RP11-357P20 for DERE). The BAC clones were purified using a large-construct DNA kit (Qiagen) and labeled by nick translation using the Nick Translation kit (Vysis, Downer Groves, IL) following the manufacturer's recommendations. Briefly, 1* µ*g of the BAC clone was conjugated with either SpectrumGreen- or SpectrumRed-labeled dUTP, coprecipitated with 10X (v/v) human Cot-1 (Invitrogen), and dissolved in Hybridization Buffer (Sigma). The reaction was carried out for 8 h at 15°C and stopped by heating the sample to 70°C for 10 min. For interphase FISH, fixed cells were treated with 0.005% pepsin for digestion, following 0.5 h treatment of 1.9N HCl at room temperature for cell denaturation. Hybridization was performed overnight at 37°C with pre-hybridized labeled probes (150 ng per sample) and slides were washed in following solution: 2X SSC (37°C for 30 min), 2X SSC (room temperature for 30 min), and 1X SSC (room temperature for 30 min). Nuclei counterstained with DAPI (0.1* µ*g/mL) were placed on a polished concave slide with Vectashield Mounting Medium (Vector Laboratories, Burlingame CA).

Images were captured by Olympus IX83 fluorescence microscope and analyzed with CellSens Dimension Imaging System. CellSens Dimension Imaging System (Olympus) was used to analyze colocalization of DERE and *THRAP1* spots in 100 cells per treatment. There are around 40-50 copies of DERE and 10-12 copies of *THRAP1*. Colocalization between the two loci was defined based on pixel overlap.

## Results

### Hormone effects on the global distribution of interactions

We report the analysis of Hi-C data from MCF-7 breast cancer cells: before (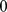) and after 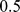, 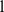, 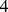 and 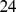 h of E2 stimulation. We first focus on long-range interactions (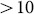 kb) obtained before binning and iterative correction. After E2 stimulation, an increase in the number of all interactions (intra- and interchromosomal) was observed peaking at 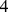 h (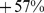) and then declining by 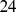 h to a value lower than the baseline (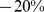, Figure S4a in File S1). Similar trends are observed using 3C-qPCR, for two randomly choosen pairs of regions (Figure S4b in File S1). Although it has been previously shown using another genome-wide technique (ChIA-PET) that E2 drives chromatin interactions in MCF7 cells [19], our results further demonstrate a more pronounced effect of E2 specifically on long-range interactions which are known to play a role in regulating gene expression.

We then binned for several resolutions (500 kb, 1 Mb, 2 Mb and 4 Mb) and iteratively corrected heatmaps for removing biases [22]. In the following, all results are based on this data preprocessing. We then used a simple and global measure - the entropy - to summarize the spatial dispersion of the distribution: the higher the entropy the higher is the spatial distribution. As shown in Figure S4c in File S1, the heatmap shows a global increase of entropy at 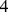 h (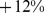) followed by a decrease at 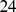 h (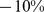). Surprisingly, this trend is essentially similar for both intra- and interchromosomal interactions, as well as for all individual chromosomes, implying that E2, not only increases the number of long-range interactions, but also leads to a global spatial reorganization of interactions over the time. At 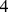 h, interactions were more widely distributed over the genome, and conversely at 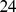 h. At the same time we observe that the average distance between interacting positions decreased from 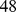 Mb to 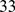 Mb at 4 h and then increased to 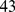 Mb at 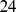 h (Figure S4d in File S1). Based on these results, we conclude that E2 induces interactions which are more spatially spread-out although located between closer positions. To better understand the above global changes in interaction distribution, we tested several possibilities, including dynamic of chromosome territories, polymer behavior and/or chromatin compartmentalization.

### Chromosome territories

Interchromosomal interaction frequencies between pairs of chromosomes show that E2 has an effect on chromosome territories (Figure S5a in File S1). After E2 stimulation, especially at 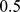 and 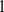 h, higher interaction frequencies are observed between small, gene-rich chromosomes (chromosomes 16, 17, 19, 20, 21, and 22), compared to the other chromosomes. This higher colocalization of small, gene-rich chromosomes provides opportunities for potentially functional interactions and facilitates sharing of subnuclear sites enriched in RNA polymerase II and other components of the transcription and RNA-processing machinery [5].

Hu *et al.* observed colocalization between E2 induced genes *TFF1* (chromosome 21) and *GREB1* (chromosome 2) [53], whereas Kocanova *et al.* did not [54]. Analysis of our Hi-C data shows that, at 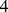 h, there is a higher frequency of interactions between the two loci (10 Mb window) surrounding the two genes (Figure S5b in File S1).

### Polymer behavior

**Figure 1:**
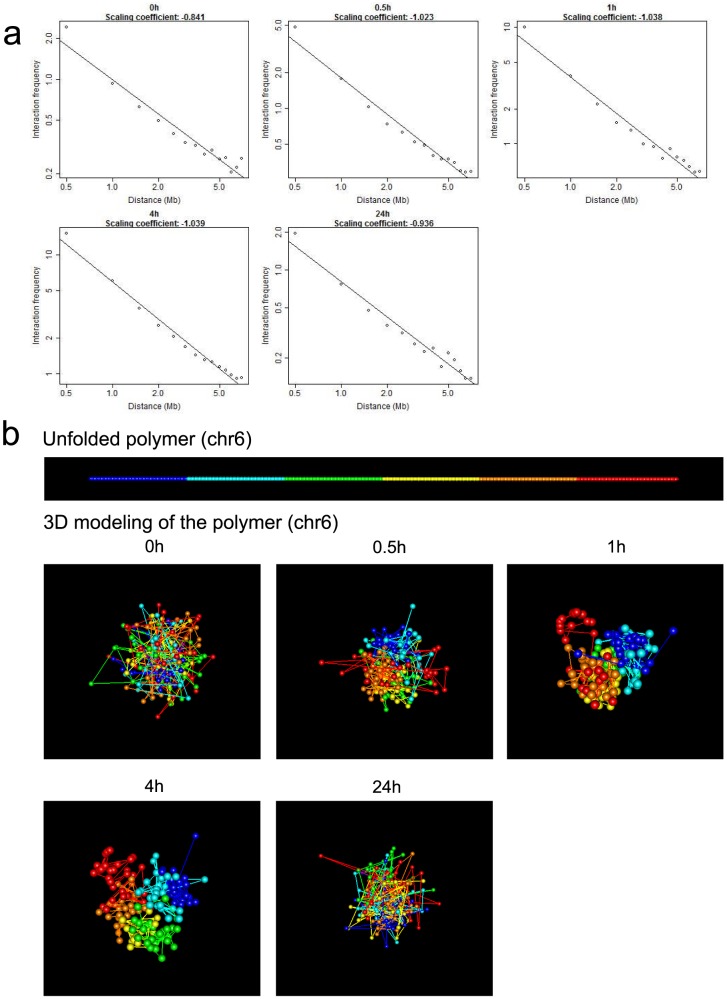
Influence of E2 on the polymer behaviour and folding of chromosomes. a) Power law dependency between intrachromosomal interaction frequency 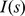 and distance 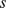. Interval 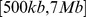, 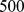 kb binning. b) 3D modeling of chromosome 6.

We then studied the effect of E2 on the polymer behavior of chromosomes. For this purpose, we calculated the scaling coefficient of intrachromosomal interaction frequency 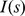 with the distance 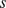 between two positions. This is done by computing a log-log regression for distances between 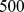 kb and 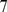 Mb, which correspond to the known size of open and closed chromatin. The analysis of our time-series data revealed that E2 influences the scaling coefficient which starts from 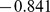 before E2 treatment, gradually increases to 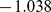 at 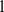 h and 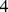 h post-E2 and then decreases to 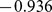 at 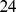 h (Figure 1a; scaling averaged over all chromosomes). Following the recent strings and binders switch (SBS) model [55], this scaling coefficient increase at 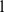 h and 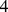 h reflects transient change from a more compact model to the fractal model, known to facilitate gene expression regulation.

To better understand the effects of E2 on chromosome folding, we modeled the 3D polymer structure of chromatin with the recent BACH program [27]. We illustrate with chromosome 6, but similar results are observed with all chromosomes. Figure 1b displays the effect of E2 on the chromosome folding for each time point. After E2 stimulation, we report a change in chromatin folding. The chromosome is less compact at 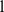 h and 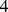 h. This observation of lower compactness confirms previous microscopic studies showing that there is an increase of chromosome territories after E2 stimulation [54]. At the same time, we do not observe significant change in nuclear volume (Figure S6 in File S1). Less compact chromosomes (i.e. chromosomes occupying a larger volume) within the same size nucleus might explain the reduced distance between interacting regions (lower average distance, Figure S4d in File S1).

### Chromatin compartmentalization

It has been previously shown that at the megabase scale chromatin segregates into two spatial compartments A and B corresponding respectively to open and closed chromatin [7]. We assessed if this compartmentalization of chromatin evolves after E2 stimulation in MCF-7 cells. For this purpose, we followed a similar methodology as in [7]. We used 1 Mb binning to estimate intrachromosomal interaction matrices with sufficient power and utilized these interaction matrices to calculate correlation matrices. These matrices reveal that, after E2 stimulation, interactions are more organized into blocks until 4 h. We illustrate with chromosome 6 (Figure S7a in File S1). Then correlation matrices were used as input for principal component analysis (PCA) to infer spatial chromatin compartments. The first component of the PCA indicates the compartment for each chromosome position. Positive values define one compartment, negative values define the other. PCA shows that E2 treatment results in a gradual increase of compartmentalization until 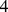 h, followed by a decline by 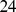 h post-E2 (Figure S7b in File S1).

**Figure 2:**
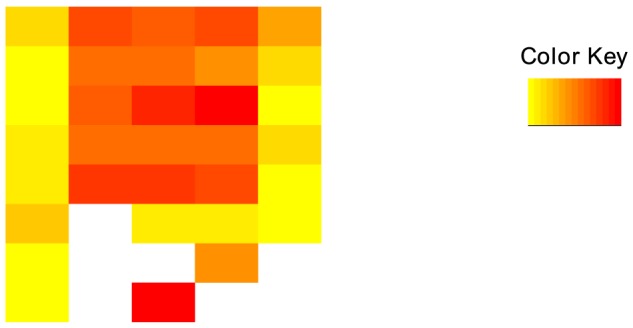
Influence of E2 on the compartmentalization of genetic and epigenetic regions. Correlation (absolute value) between compartmentalization and genetic and epigenetic marks, for the chromosome 6. For a better visualization, row values have been scaled (Z-score).

To interpret the chromatin compartments, we calculated Pearson correlations between the compartment status and genetic and epigenetic features of the genome over the time (Figure 2). The biological meaning of the correlation is straightforward: assessing spatial closeness of chromatin positions sharing a similar genetic or epigenetic pattern. At baseline, correlations with features are low (between 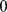 and 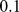) and significantly increase after E2 treatment, reaching a maximum at 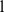 h and 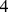 h (between 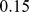 and 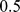, except for gene expression). At 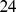 h, correlations were similar to baseline values. These results are confirmed by 3D modeling of chromatin (Figure S8 in File S1). Chromatin folding reveals that gene concentration, H3K4Me2, DNA methylation, CTCF, DNaseI HS, RNA polymerase II and ER*α* binding, colocalize with compartment A. Absence of colocalization with microarray gene expression data is not surprising given the noisy nature of such data and the scale of analysis (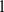 Mb). From these results, we conclude that E2 induces a higher spatial compartmentalization of active and repressive marked, gene rich, highly organized, expressed, and open chromatin regions.

### Interacting loci analysis

We next investigated the function of E2-induced differential interactions between genomic loci. For this purpose, we first assessed functional enrichments to draw a global picture. Differentially interacting loci were calculated for any time point t (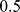, 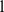, 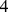 or 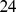 h) against time point 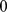 h, using the Wilcoxon test for paired samples with a resolution of 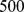 kb. Active genes were mapped within the interacting loci. Then DAVID program was used to determine clusters of enriched gene ontology (GO) terms. The 100 most differentially interacting loci were analyzed for each time point (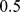, 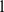, 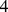 or 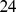 h). From these differentially interacting loci, 183, 293, 353 and 210 genes were mapped at 0.5, 1, 4 and 24 h, respectively (Table 1). This reveals a key result: regions richer in genes are more differentially interacting due to E2 induction, reaching a peak at 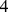 h. Moreover functional enrichment analysis shows that most enriched GO terms are known to be affected by estrogen and/or related to cancer progression. As a good example, the term “apoptosis” which represents a key component of cancer proliferation is reported from 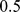 h until 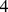 h. Except “apoptosis” and few others, most terms change over the time. They reflect the dynamics of functional chromatin conformations after E2 stimulation. For instance, terms such as “contractile fiber”, “G protein signalling”, “skeletal system development” and “cell adhesion” are only enriched at 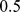 h. An interesting enriched term is “citrullination” observed from 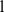 to 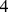 h. The differentially interacting locus contains the PAD gene family (*PAD1*, *PAD2*, *PAD3*, *PAD4* and *PAD6*). It has recently been reported that ER*α* interacts with *PAD2* and that *PAD2*-mediated citrullination leads to local chromatin decondensation and transcriptional activation of target genes [56]. Other interesting GO terms found at 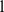 and 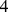 h are “nucleosome”, “chromatin organization” and “chromatin remodeling”. They are crucial processes enabling ER*α* to bind to estrogen responsive elements (EREs) [57]. At 24 h after E2 stimulation, most GO terms are new and reflect late gene response. Among those terms, regulation of cell growth represents a major mechanism for cancer proliferation. It is also worth mentioning the term “response to steroid hormone stimulus”, since it supports the biological relevance of our E2-induced chromatin interactions analysis.

**Table 1:** Functional enrichment clusters of differentially interacting genes after E2 stimulation.

Time point	Number of genes	Annotation cluster	Enrichment score
0.5 h	183	Neurotransmitter transport	1.85
		Stem cell differentiation	1.73
		Contractile fiber	1.28
		Apoptosis	1.27
		Amino acid transport	1.21
		G protein signalling	1.2
		Isopeptide bond	1.15
		Immune system development	1.09
		Lumen	0.91
		Skeletal system development, cell adhesion	0.87
1 h	293	Citrullination	3.83
		Nucleosome and chromatin organization	1.87
		Chromatin remodeling	1.33
		Peroxisome	1.23
		Induction of apoptosis	1.08
		Regulation of GTPase activity	1.04
		Protein transport and localization	1.04
		EGF-like domain	1.03
		Regulation of kinase activity	0.97
		WW domain	0.94
4 h	275	Citrullination	5.02
		Nucleosome and chromatin organization	1.84
		F-box domain	1.64
		Tetraspanin	1.25
		Proteolysis	1.01
		Apoptosis	0.95
		Protein transport and localization	0.91
		RNA transport	0.9
		Cell cycle and cytoskeleton	0.87
		Ribosome	0.79
24 h	138	Neurotransmitter transport	1.58
		GTPase binding	1.48
		HEAT repeat domain	1.23
		Regulation of cell growth	1.23
		Lumen	1.19
		GTPase activity	1.02
		WD repeat domain	1
		PDZ domain	0.93
		Neuron differentiation	0.8
		Response to steroid hormone stimulus	0.77

**Figure 3:**
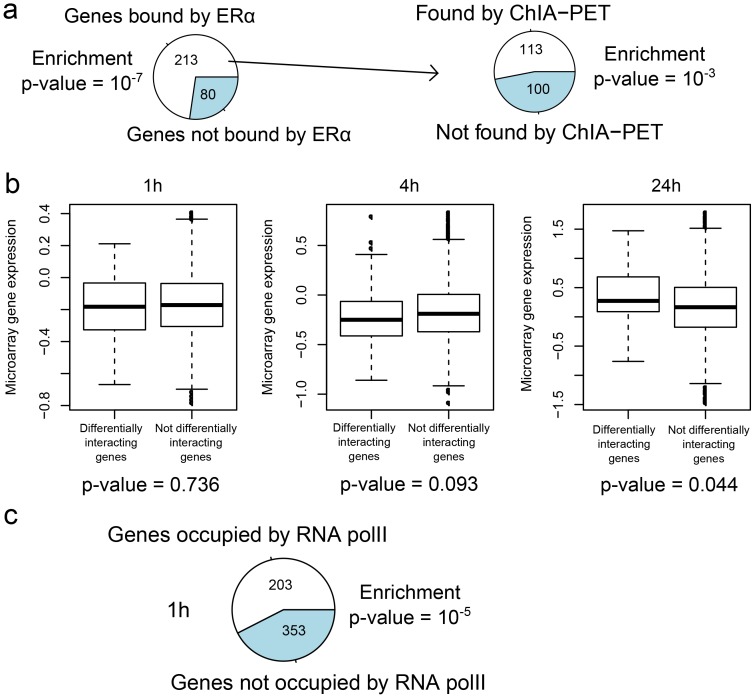
Link between differential interaction and ER*α* binding and gene transcription. a) Enrichment analysis of differentially interacting genes with ER*α* binding and comparison with ChIA-PET results. b) Expression of differentially versus non-differentially interacting genes. c) Enrichment analysis of differentially interacting genes with RNA polymerase II occupancy.

We further assessed the link between differential interaction loci genes and ER*α* binding, and gene expression. Of the 293 differentially interacting loci genes at 1 h, 213 were bound by ER*α* which represents a significant enrichment (binomial enrichment p-value  =  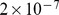, Figure 3a). Among these 213 differentially interacting and ER*α* bound loci genes, 113 were reported using ChIA-PET (binomial enrichment p-value  =  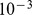, Figure 3a). These results indicate that most differentially interacting loci genes involves ER*α* binding. Besides, validation with ChIA-PET results reveals that combining Hi-C data with ER*α* binding represents an efficient approach to identify biologically relevant interactions. Regarding gene regulation, microarray data show no differences of expression between differentially interacting loci genes and non-differentially interacting loci genes at 1 h (p-value  = 0.736) and 4 h (p-value  = 0.093), and slight differences at 24 h (p-value  = 0.044) (Figure 3b). As for compartmentalization analysis (previous subsection), these negative results are not conclusive given the noisy nature of microarray data and the scale of analysis (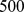 kb). We then explored the correlation between differentially interacting loci genes and RNA polymerase II occupancy, more accurate to detect differences in the trancriptional process. Enrichment analysis points out a signicantly higher RNA polymerase II occupancy in differentially interacting loci genes compared to non-differentially interacting loci genes (p-value  =  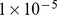, Figure 3c). Taken together these results show that differentially interacting loci genes are enriched for ER*α* binding events and are correlated with transcriptional process.

### Interacting loci network

**Figure 4:**
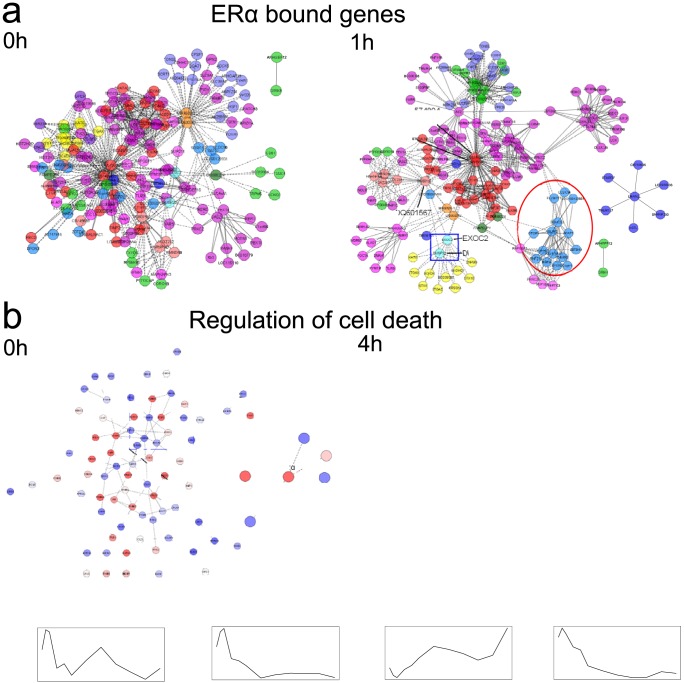
Influence of E2 on the network of interacting genes. a) Networks of ER*α* bound interacting genes. Each color represents a different chromosome. The red circle highlights a cluster of nodes belonging to the same chromosome. The blue frame highlights a hub connecting different chromosomes. b) Networks of interacting genes belonging to the GO term “regulation of cell death” (GO:0010941). Blue nodes denote low expression, while red nodes represent high expression. Blue frames are zooms inside the networks. For the sake of graphical display, only interacting nodes are shown in both Figures 4a and 4b. Straight lines are interchromosomal interactions, dashed lines are interchromosomal interactions. c) Expression of genes TGFBR2, EGR1, DAXX and BCL2L1.

Interacting gene networks were built using CytoHiC with a resolution of 500 kb [25]. We first investigated interacting genes involved in ER*α* binding events to better understand the role of ER*α*-mediated E2 effect on functional chromatin interactions and conformation. The integration of ER*α* bound genes with Hi-C data reveals E2 effect on the network of interactions (Figure 4a). After E2 stimulation, the network shows a less centralized configuration: network centralization coefficient is reduced from 0.449 (0 h) to 0.238 (1 h). At 1 h, the genes belonging to the same chromosome (sharing same colors in the graph) are more closely connected. For instance genes from chromosome 4 (blue nodes encircled in red) are clustered. Moreover some nodes connect genes belonging to different chromosomes. For example, the *EXOC2* and *DUSP22* genes from chromosome 6 (light blue nodes framed in blue) connect a group of genes from chromosome 6 (yellow nodes) to the *DQ601567* gene from chromosome 5, and to the *DQ574804* and *DQ575686* genes from chromosome 17. This change of interacting gene network configuration reveals the role of ER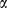 as a chromatin interaction organizer. After E2 stimulation, ER*α* enables higher interactions among genes of the same chromosome, while maintaining interchromosomal interactions through hub nodes connecting chromosomes.

We then sought to assess whether E2 induces changes in interacting networks of genes involved in a known E2-related biological process such as “regulation of cell death” (GO:0010941). E2 is a potent inhibitor of apoptosis and it regulates the expression of several apoptotic proteins [58]. Figure 4b displays the interacting gene networks. Before E2, we observe that *ERα* gene interacts with two tumor suppressor genes (*EGR1* and *TGFBR2*). However after E2 stimulation, these genes don't interact anymore with *ERα* and their expressions are inhibited (Figure 4c). This finding is consistent with a recently proposed mechanism wherein E2-mediated repression of genes is due to chromatin structure reconfiguration, thereby disrupting the originally transcription-efficient chromatin structures [37]. At 4 h after E2 stimulation, *ERα* gene interacts with two apoptotic genes *DAXX* [59] and *BCL2L1* [60]. The interactions of apoptotic genes with *ERα* is associated with changes of expressions. *DAXX* expression is induced, while the opposite trend is reported for *BCL2L1* (Figure 4c). These observations suggest that E2-responsive genes are not only regulated through ER*α* protein but also by interacting with *ERα* gene (such as with its promoter), and this can be a pathway to regulate cancer proliferation.

### Analysis of 17q23 and 20q13 loci

**Figure 5:**
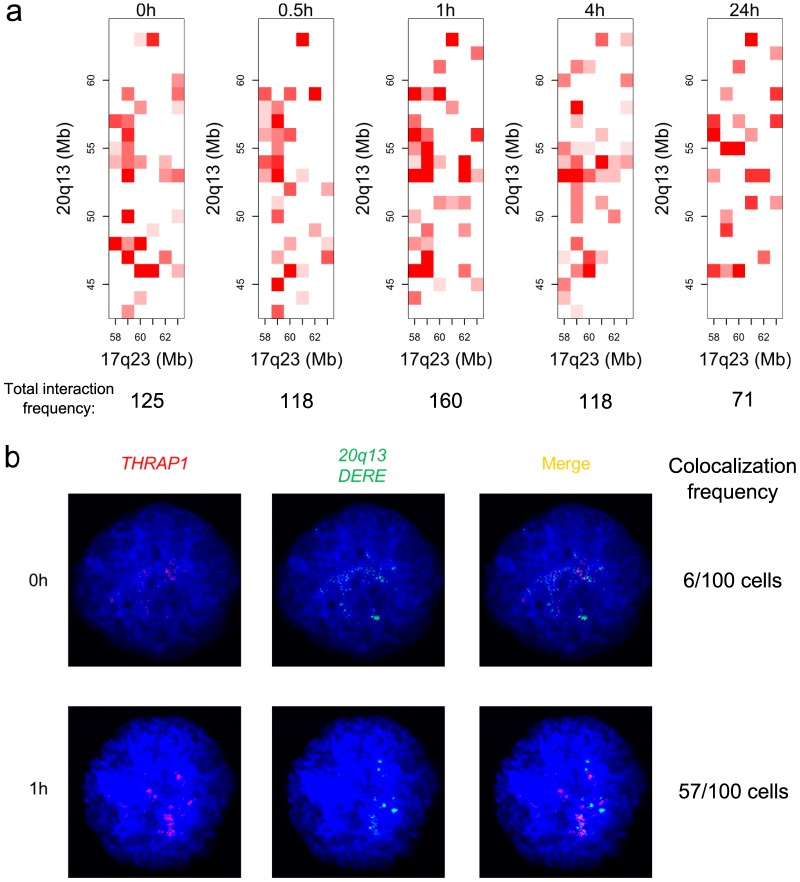
Influence of E2 on the spatial proximity between the 17q23 and 20q13 loci. a) Hi-C interaction heatmap. b) Interphase FISH analysis of THRAP1 [chr17:60019966-60142643] and 20q13 DERE [chr20:54155758-54155858].

Two densely mapped distant estrogen responsive elements (DEREs) located in 17q23 and 20q13 loci have been recently shown to be involved in frequent amplification in MCF-7 cells. These DEREs remotely control the transcription of target genes present on different chromosomes through chromatin proximity [36]. However an important issue remains to know if these loci interact with each other for a better coregulation of target genes, and the sharing of transcriptional machinery. To tackle this issue, we investigated interactions between the two loci using Hi-C data (Figure 5a). The heatmaps show an increase of interaction frequencies at 1 h (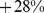), followed by a decrease until 24 h (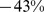). This result is supported by interphase FISH analysis between *THRAP1* (located in 17q23) and DERE from 20q13 (Figure 5b). After E2 stimulation, there is a dramatic increase of colocalization events of the two loci at 1 h (57/100 colocalization events, compared to 6/100 colocalization events at 0 h). For addressing the specificity of DERE-*THRAP1* colocalization, a control gene, *GAPDH*, was used. No colocalization events were found at 1 h between DERE and *GAPDH* or between DERE and *THRAP1* (Figure S9 in File S1). Similar interaction results between *THRAP1* and 20q13 DERE were reported using 3C-qPCR analysis [36]. Besides functional interplay of the two loci, their interactions provide an explanation for the observed high number of fusion events between the two loci (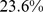 of all fusions over the genome) [36], since spatial proximity influences chromosomal rearrangements [61]. The interaction analysis suggests that E2-induced proximity between the two loci might be a potential mechanism by which fusion events arise in ER-positive breast cancer cells.

## Conclusion

Our work shows that time-series Hi-C data analysis is a promising methodology for studying the global dynamic of chromatin and its impact on gene regulation. Our results reveal a dramatic structural effect of a hormone on genome folding, meaning that not only chromatin can be locally modified through loop formations but also the whole genome organization can be rearranged in a relatively short amount of time.

To better understand the impact of E2 on genome organization, chromosome territories, polymer-like behavior and chromatin compartmentalization were studied. Analysis of interchromosomal interactions reveals that small gene-rich chromosomes (chromosomes 16-17 and 19–22) tend to interact more with each other after E2 stimulation. At the chromosome level, E2 affects the polymer-like behavior of chromatin from a more compact to a fractal model, thus facilitating gene regulation. Three dimension modeling shows a higher organization of chromatin compartmentalization where linear positions are in closer spatial proximity.

To biologically interpret this global change of genome organization, Hi-C data were integrated with ER*α* binding, gene expression and multiple epigenetic marks. For some chromosomes, such as chromosome 6, E2 induces a higher spatial compartmentalization of active and repressive marked, gene rich, highly organized and open chromatin regions. At a smaller scale, we observe that differential chromatin interactions are mostly localized in gene-rich regions after E2 stimulation. Moreover, most interacting genes are enriched for gene ontology terms known to be affected by estrogen and/or related to cancer progression. ER*α* binding is significantly associated with a large part of these interacting genes. Regarding expression, differential interactions appear to be correlated with regulation of gene transcription. Network analysis show that E2 induces interactions between *ERα* gene and apoptotic genes, which can be a pathway for E2 to regulate cancer cell proliferation through promoter-promoter interactions. In addition, analysis of interactions between DEREs located in 17q23 and 20q13 loci reveals their E2-induced spatial proximity and suggests coregulation of target genes over the genome. This E2-induced spatial proximity also provides an explanation for the observed high number of fusion events between the two loci.

Hi-C interactions were validated using other techniques. For instance, Hi-C interactions between 17q23 and 20q13 at 1 and 4 h were confirmed by spatial proximity using FISH analysis. Similar interaction results between the two loci were reported using 3C-qPCR analysis [36]. In addition, a large number of our integrated Hi-C analysis results were also found by ChIA-PET. This demonstrates that combination of Hi-C data with ER*α* binding events represents an efficient approach to identify biologically relevant interactions. It is also worth noting that our Hi-C results make precise the large scale chromatin organization change previously observed using microscopy [38].

These results are promising but there is still much to be done. Although the recent strings and binders switch model can explain the observed changes in polymer-like behavior of chromatin, it is not clear what it is the biological mechanism behind these changes. Regarding the functional relevance of interactions, our work only pointed out correlations between differential interactions, ER*α* binding events and expression, but molecular functional studies will be required to reveal causality relations. Finally this work provides a global − 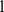 Mb - view of genome organization change after E2 stimulation and its role in gene expression regulation. To be able to focus at a smaller scale, further studies should increase the number of reads. This would help assess the potential effect of E2 on the topogically associating domains. Moreover, local changes through loop formation have also an important role in the regulation of expression of ER-dependent genes. For these ER-dependent genes, chromatin interactions play as the initiating step for bringing transcription complex binding onto the target genes, leading to expression alteration. This explains why ER-dependent gene expression remains high even at 24 hr after E2 stimulation (Figures S1 and S2 in File S1). Our ongoing study using 3C-ChIP-qPCR assay further supports this statement by showing that de novo loop formation occurs first, following recruitment of repressive histone marks binding onto the looping event for suppressing expression of the examined target gene.

## Supporting Information

File S1 Figures S1-S9 and Table S1.
(PDF)

## References

[1] Meaburn KJ, Misteli T (2007) Chromosome territories. Cell Biology 445:379–381. 10.1038/445379a 17251970

[2] Lanctôt C, Cheutin T, Cremer M, Cavalli G, Cremer T (2007) Dynamic genome architecture in the nuclear space: regulation of gene expression in three dimensions. Nature Reviews Genetics 8:104–115. 10.1038/nrg2041 17230197

[3] Sexton T, Schober H, Fraser P, Gasser SM (2007) Gene regulation through nuclear organization. Nature Structural & Molecular Biology 14:1049–1055. 10.1038/nsmb1324 17984967

[4] Fudenberg G, Getz G, Meyerson M, Mirny LA (2011) High order chromatin architecture shapes the landscape of chromosomal alterations in cancer. Nature Biotechnology 29:1109–1113. 10.1038/nbt.2049 PMC3268007 22101486

[5] Dekker J, Marti-Renom MA, Mirny LA (2013) Exploring the three-dimensional organization of genomes: interpreting chromatin interaction data. Nature Reviews Genetics 14:390–403. 10.1038/nrg3454 PMC3874835 23657480

[6] Simonis M, Klous P, Splinter E, Moshkin Y, Willemsen R, et al (2006) Nuclear organization of active and inactive chromatin domains uncovered by chromosome conformation capture-on-chip (4C). Nature Genetics 38:1348–1354. 17033623 10.1038/ng1896

[7] Lieberman-Aiden E, van Berkum NL, Williams L, Imakaev M, Ragoczy T, et al (2009) Comprehensive mapping of long-range interactions reveals folding principles of the human genome. Science 326:289–293. 19815776 10.1126/science.1181369 PMC2858594

[8] Sexton T, Yaffe E, Kenigsberg E, Bantignies F, Leblanc B, et al (2012) Three-dimensional folding and functional organization principles of the Drosophila genome. Cell 148:458–472. 22265598 10.1016/j.cell.2012.01.010

[9] Mirny LA (2011) The fractal globule as a model of chromatin architecture in the cell. Chromosome Research 19:37–51. 21274616 10.1007/s10577-010-9177-0 PMC3040307

[10] Fudenberg G, Mirny LA (2012) Higher-order chromatin structure: Bridging physics and biology. Current Opinion in Genetics & Development 22:1–10. 22360992 10.1016/j.gde.2012.01.006 PMC3697851

[11] Dixon JR, Selvaraj S, Yue F, Kim A, Li Y, et al (2012) Topological domains in mammalian genomes identified by analysis of chromatin interactions. Nature 485:376–380. 22495300 10.1038/nature11082 PMC3356448

[12] Lan X, Witt H, Katsumura K, Ye Z, Wang Q, et al (2012) Integration of Hi-C and ChIP-seq data reveals distinct types of chromatin linkages. Nucleic Acids Research 40:7690–7704. 22675074 10.1093/nar/gks501 PMC3439894

[13] Botta M, Haider S, Leung IX, Lio P, Mozziconacci J (2010) Intra- and inter-chromosomal interactions correlate with CTCF binding genome wide. Molecular Systems Biology 6:426. 21045820 10.1038/msb.2010.79 PMC3010120

[14] van Berkum NL, Lieberman-Aiden E, Williams L, Imakaev M, Gnirke A, et al (2010) Hi-C: a method to study the three-dimensional architecture of genomes. Journal of Visualized Experiments 39:1869. 10.3791/1869 PMC3149993 20461051

[15] Dekker J, Rippe K, Dekker M, Kleckner N (2002) Capturing chromosome conformation. Science 295:1306–1311. 11847345 10.1126/science.1067799

[16] Zhao Z, Tavoosidana G, Sjölinder M, Göndör A, Mariano P, et al (2006) Circular chromosome conformation capture (4C) uncovers extensive networks of epigenetically regulated intra- and interchromosomal interactions. Nature Genetics 38:1341–1347. 17033624 10.1038/ng1891

[17] Dostie J, Richmond TA, Arnaout RA, Selzer RR, Lee WL, et al (2006) Chromosome Conformation Capture Carbon Copy (5C): A massively parallel solution for mapping interactions between genomic elements. Genome Research 16:1299–1309. 16954542 10.1101/gr.5571506 PMC1581439

[18] Kalhor R, Tjong H, Jayathilaka N, Alber F, Chen L (2012) Genome architectures revealed by tethered chromosome conformation capture and population-based modeling. Nature Biotechnology 30:90–98. 10.1038/nbt.2057 PMC3782096 22198700

[19] Fullwood MJ, Liu MHH, Pan YFF, Liu J, Xu H, et al (2009) An oestrogen-receptor-alpha-bound human chromatin interactome. Nature 462:58–64. 19890323 10.1038/nature08497 PMC2774924

[20] Li G, Ruan X, Auerbach RK, Sandhu KSS, Zheng M, et al (2012) Extensive promoter-centered chromatin interactions provide a topological basis for transcription regulation. Cell 148:84–98. 22265404 10.1016/j.cell.2011.12.014 PMC3339270

[21] Yaffe E, Tanay A (2011) Probabilistic modeling of Hi-C contact maps eliminates systematic biases to characterize global chromosomal architecture. Nature Genetics 43:1059–1065. 22001755 10.1038/ng.947

[22] Imakaev M, Fudenberg G, McCord RP, Naumova N, Goloborodko A, et al (2012) Iterative correction of Hi-C data reveals hallmarks of chromosome organization. Nature Methods 9:999–1003. 22941365 10.1038/nmeth.2148 PMC3816492

[23] Cournac A, Marie-Nelly H, Marbouty M, Koszul R, Mozziconacci J (2012) Normalization of a chromosomal contact map. BMC Genomics 13:436. 22935139 10.1186/1471-2164-13-436 PMC3534615

[24] Hu M, Deng K, Selvaraj S, Qin Z, Ren B, et al (2012) HiCNorm: removing biases in Hi-C data via Poisson regression. Bioinformatics 28:3131–3133. 23023982 10.1093/bioinformatics/bts570 PMC3509491

[25] Shavit Y, Lio' P (2013) CytoHiC: a cytoscape plugin for visual comparison of Hi-C networks. Bioinformatics 29:1206–1207. 23508968 10.1093/bioinformatics/btt120

[26] Rousseau M, Fraser J, Ferraiuolo M, Dostie J, Blanchette M (2011) Three-dimensional modeling of chromatin structure from interaction frequency data using Markov chain Monte Carlo sampling. BMC Bioinformatics 12:414. 22026390 10.1186/1471-2105-12-414 PMC3245522

[27] Hu M, Deng K, Qin Z, Dixon J, Selvaraj S, et al. (2013) Bayesian inference of spatial organizations of chromosomes. PLoS Computational Biology 9: e1002893+. 10.1371/journal.pcbi.1002893 PMC3561073 23382666

[28] Gasser SM (2002) Visualizing chromatin dynamics in interphase nuclei. Science 296:1412–1416. 12029120 10.1126/science.1067703

[29] Hübner MR, Spector DL (2010) Chromatin dynamics. Annual Review of Biophysics 39:471–489. 10.1146/annurev.biophys.093008.131348 PMC2894465 20462379

[30] Cavalli G (2007) Chromosome kissing. Current Opinion in Genetics & Development 17:443–450. 17933509 10.1016/j.gde.2007.08.013

[31] Gerlich D, Beaudouin J, Kalbfuss B, Daigle N, Eils R, et al (2003) Global chromosome positions are transmitted through mitosis in mammalian cells. Cell 112:751–764. 12654243 10.1016/s0092-8674(03)00189-2

[32] Carroll JS, Meyer CA, Song J, Li W, Geistlinger TR, et al (2006) Genome-wide analysis of estrogen receptor binding sites. Nature Genetics 38:1289–1297. 17013392 10.1038/ng1901

[33] Hua S, Kallen CB, Dhar R, Baquero MT, Mason CE, et al (2008) Genomic analysis of estrogen cascade reveals histone variant H2A.Z associated with breast cancer progression. Molecular Systems Biology 4:188. 18414489 10.1038/msb.2008.25 PMC2394496

[34] Fan M, Yan PS, Hartman-Frey C, Chen L, Paik H, et al (2006) Diverse gene expression and DNA methylation profiles correlate with differential adaptation of breast cancer cells to the antiestrogens tamoxifen and fulvestrant. Cancer Research 66:11954–11966. 17178894 10.1158/0008-5472.CAN-06-1666

[35] Hsu PY, Hsu HK, Singer GA, Yan PS, Rodriguez BA, et al (2010) Estrogen-mediated epigenetic repression of large chromosomal regions through DNA looping. Genome Research 20:733–744. 20442245 10.1101/gr.101923.109 PMC2877570

[36] Hsu PY, Hsu HK, Lan X, Juan L, Yan PS, et al (2013) Amplification of distant estrogen response elements deregulates target genes associated with tamoxifen resistance in breast cancer. Cancer Cell 24:197–212. 23948299 10.1016/j.ccr.2013.07.007 PMC3890247

[37] Osmanbeyoglu HU, Lu KN, Oesterreich S, Day RS, Benos PV, et al (2013) Estrogen represses gene expression through reconfiguring chromatin structures. Nucleic Acids Research 41:8061–8071. 23821662 10.1093/nar/gkt586 PMC3783169

[38] Nye AC, Rajendran RR, Stenoien DL, Mancini MA, Katzenellenbogen BS, et al (2002) Alteration of large-scale chromatin structure by estrogen receptor. Molecular and Cellular Biology 22:3437–3449. 11971975 10.1128/MCB.22.10.3437-3449.2002 PMC133805

[39] Rickman DS, Soong TD, Moss B, Mosquera JMM, Dlabal J, et al (2012) Oncogene-mediated alterations in chromatin conformation. Proceedings of the National Academy of Sciences of the United States of America 109:9083–9088. 22615383 10.1073/pnas.1112570109 PMC3384175

[40] Hampton OA, Den Hollander P, Miller CA, Delgado DA, Li J, et al (2009) A sequence-level map of chromosomal breakpoints in the MCF-7 breast cancer cell line yields insights into the evolution of a cancer genome. Genome Research 19:167–177. 19056696 10.1101/gr.080259.108 PMC2652200

[41] Cowper-Sal lari R, Zhang X, Wright JB, Bailey SD, Cole MD, et al (2012) Breast cancer risk-associated SNPs modulate the affinity of chromatin for *FOXA1* and alter gene expression. Nature Genetics 44:1191–1198. 23001124 10.1038/ng.2416 PMC3483423

[42] Sabo PJ, Hawrylycz M, Wallace JC, Humbert R, Yu M, et al (2004) Discovery of functional noncoding elements by digital analysis of chromatin structure. Proceedings of the National Academy of Sciences of the United States of America 101:16837–16842. 15550541 10.1073/pnas.0407387101 PMC534745

[43] Sabo PJ, Kuehn MS, Thurman R, Johnson BE, Johnson EM, et al (2006) Genome-scale mapping of DNase I sensitivity in vivo using tiling DNA microarrays. Nature Methods 3:511–518. 16791208 10.1038/nmeth890

[44] Meissner A, Mikkelsen TS, Gu H, Wernig M, Hanna J, et al (2008) Genome-scale DNA methylation maps of pluripotent and differentiated cells. Nature 454:766–770. 18600261 10.1038/nature07107 PMC2896277

[45] Innes CR, Brown G, Carroll J (2011) A co-ordinated interaction between CTCF and ER in breast cancer cells. BMC Genomics 12:593. 22142239 10.1186/1471-2164-12-593 PMC3248577

[46] Putnik M, Zhao C, ke Gustafsson J, Dahlman-Wright K (2012) Global identification of genes regulated by estrogen signaling and demethylation in MCF-7 breast cancer cells. Biochemical and Biophysical Research Communications 426:26–32. 22902638 10.1016/j.bbrc.2012.08.007

[47] Consortium TEP (2012) An integrated encyclopedia of DNA elements in the human genome. Nature 489:57–74. 22955616 10.1038/nature11247 PMC3439153

[48] Cicatiello L, Mutarelli M, Grober OM, Paris O, Ferraro L, et al (2010) Estrogen receptor *α* controls a gene network in luminal-like breast cancer cells comprising multiple transcription factors and microRNAs. The American Journal of Pathology 176:2113–2130. 20348243 10.2353/ajpath.2010.090837 PMC2861078

[49] Wang G, Wang Y, Shen C, Huang Yw, Huang K, et al (2010) RNA polymerase II binding patterns reveal genomic regions involved in microRNA gene regulation. PLoS ONE 5:e13798. 21072189 10.1371/journal.pone.0013798 PMC2970572

[50] Welboren WJ, van Driel MA, Janssen-Megens EM, van Heeringen SJ, Sweep FCGJ, et al (2009) ChIP-Seq of ER*α* and RNA polymerase II defines genes differentially responding to ligands. The EMBO Journal 28:1418–1428. 19339991 10.1038/emboj.2009.88 PMC2688537

[51] Lee TI, Johnstone SE, Young RA (2006) Chromatin immunoprecipitation and microarray-based analysis of protein location. Nature Protocols 1:729–748. 17406303 10.1038/nprot.2006.98 PMC3004291

[52] Hagège H, Klous P, Braem C, Splinter E, Dekker J, et al (2007) Quantitative analysis of chromosome conformation capture assays (3C-qPCR). Nature Protocols 2:1722–1733. 17641637 10.1038/nprot.2007.243

[53] Hu Q, Kwon YS, Nunez E, Cardamone MD, Hutt KR, et al (2008) Enhancing nuclear receptor-induced transcription requires nuclear motor and *LSD1*-dependent gene networking in interchromatin granules. Proceedings of the National Academy of Sciences of the United States of America 105:19199–19204. 19052240 10.1073/pnas.0810634105 PMC2593616

[54] Kocanova S, Kerr EA, Rafique S, Boyle S, Katz E, et al (2010) Activation of estrogen-responsive genes does not require their nuclear co-localization. PLoS Genetics 6:e1000922. 20421946 10.1371/journal.pgen.1000922 PMC2858706

[55] Barbieri M, Chotalia M, Fraser J, Lavitas LM, Dostie J, et al (2012) Complexity of chromatin folding is captured by the strings and binders switch model. Proceedings of the National Academy of Sciences of the United States of America 109:16173–16178. 22988072 10.1073/pnas.1204799109 PMC3479593

[56] Zhang X, Bolt M, Guertin MJ, Chen W, Zhang S, et al (2012) Peptidylarginine deiminase 2-catalyzed histone H3 arginine 26 citrullination facilitates estrogen receptor *α* target gene activation. Proceedings of the National Academy of Sciences of the United States of America 109:13331–13336. 22853951 10.1073/pnas.1203280109 PMC3421185

[57] Belandia B, Orford RL, Hurst HC, Parker MG (2002) Targeting of SWI/SNF chromatin remodelling complexes to estrogen-responsive genes. The EMBO Journal 21:4094–4103. 12145209 10.1093/emboj/cdf412 PMC126156

[58] Perillo B, Sasso A, Abbondanza C, Palumbo G (2000) 17*β*-estradiol inhibits apoptosis in MCF-7 cells, inducing *bcl-2* expression via two estrogen-responsive elements present in the coding sequence. Molecular and Cellular Biology 20:2890–2901. 10733592 10.1128/mcb.20.8.2890-2901.2000 PMC85519

[59] Yang X, Khosravi-Far R, Chang HY, Baltimore D (1997) Daxx, a novel Fas-binding protein that activates JNK and apoptosis. Cell 89:1067–1076. 9215629 10.1016/s0092-8674(00)80294-9 PMC2989411

[60] Hsu YT, Wolter KG, Youle RJ (1997) Cytosol-to-membrane redistribution of *Bax* and *Bcl-XL* during apoptosis. Proceedings of the National Academy of Sciences of the United States of America 94:3668–3672. 9108035 10.1073/pnas.94.8.3668 PMC20498

[61] Wijchers PJ, de Laat W (2011) Genome organization influences partner selection for chromosomal rearrangements. Trends in Genetics 27:63–71. 21144612 10.1016/j.tig.2010.11.001

