# Correction: Estrogen Induces Global Reorganization of Chromatin Structure in Human Breast Cancer Cells

**DOI:** 10.1371/journal.pone.0118237

**Published:** 2015-03-18

**Authors:** The PLOS ONE Staff

Fig. 2 is incorrect. Please see the corrected Fig. 2 here.

**Fig. 2:**
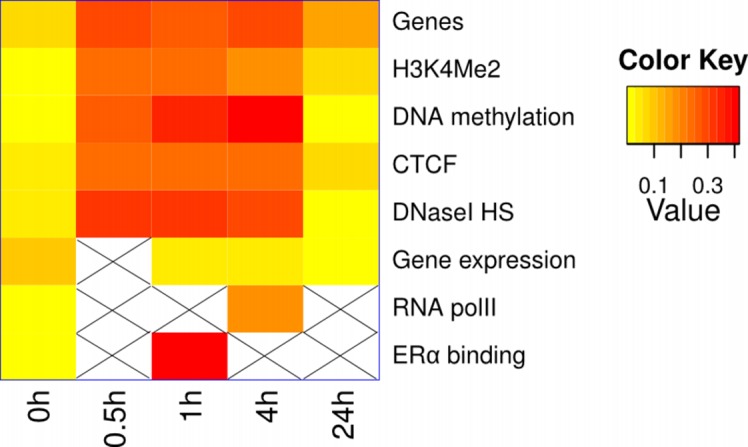
Influence of E2 on the compartmentalization of genetic and epigenetic regions. Correlation (absolute value) between compartmentalization and genetic and epigenetic marks, for the chromosome 6. For a better visualization, row values have been scaled (Z-score).

Fig. 4 is incorrect. Please see the corrected Fig. 4 here.

**Fig. 4:**
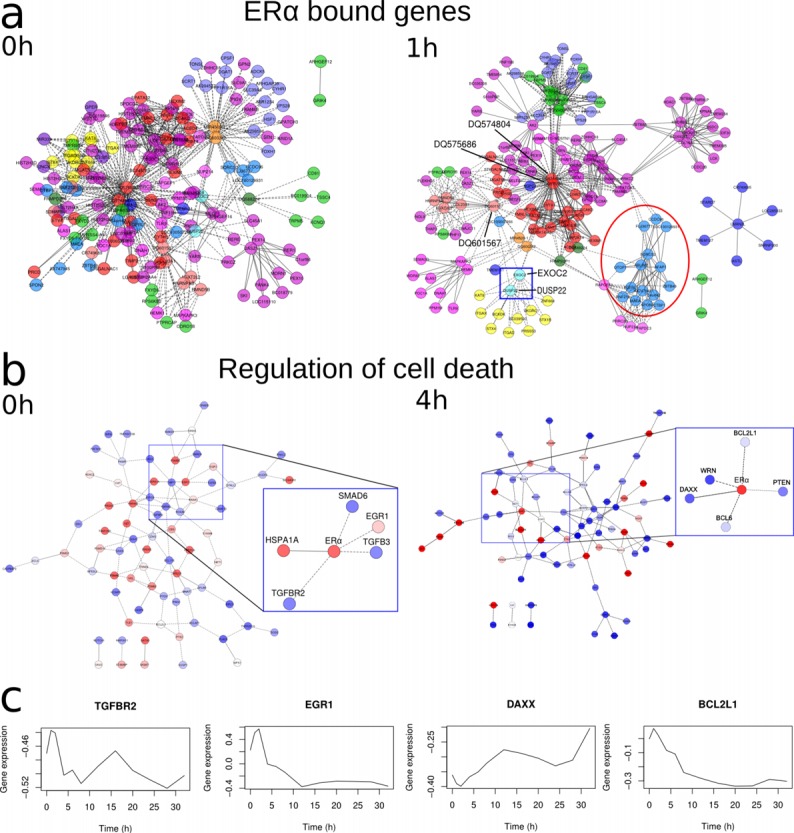
Influence of E2 on the network of interacting genes. a) Networks of ERα bound interacting genes. Each color represents a different chromosome. The red circle highlights a cluster of nodes belonging to the same chromosome. The blue frame highlights a hub connecting different chromosomes. b) Networks of interacting genes belonging to the GO term “regulation of cell death” (GO:0010941). Blue nodes denote low expression, while red nodes represent high expression. Blue frames are zooms inside the networks. For the sake of graphical display, only interacting nodes are shown in both Figures 4a and 4b. Straight lines are interchromosomal interactions, dashed lines are interchromosomal interactions. c) Expression of genes TGFBR2, EGR1, DAXX and BCL2L1.
