# Supplementary material for: Estrogen Induces Global Reorganization of Chromatin Structure in Human Breast Cancer Cells
Source: PLoS One. 2014 Dec 3;9(12):e113354. doi: 10.1371/journal.pone.0113354 (PMC4255042; doi:10.1371/journal.pone.0113354)
Supplement: File S1 — Figures S1-S9 and Table S1. (PDF) [file pone.0113354.s001.pdf]

File S1

Supporting information file S1 includes Figures S1-S9 and Table S1.

Figure S1

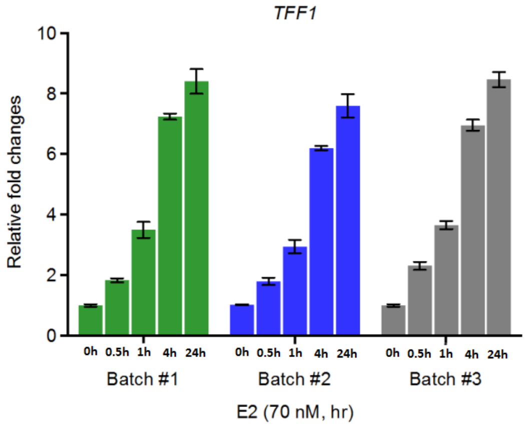

Expression analysis of *TFF1* in three different batches of cells by RT-qPCR.

Figure S2

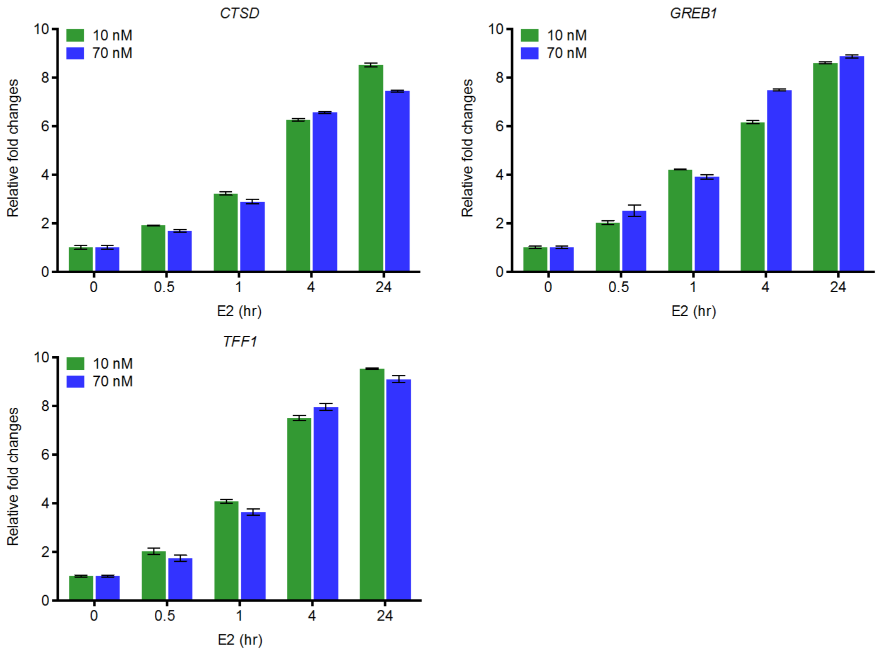

Expression analysis of three estrogen-responsive genes (*CTSD*, *GREB1* and *TFF1*) by RT-qPCR.

Figure S3

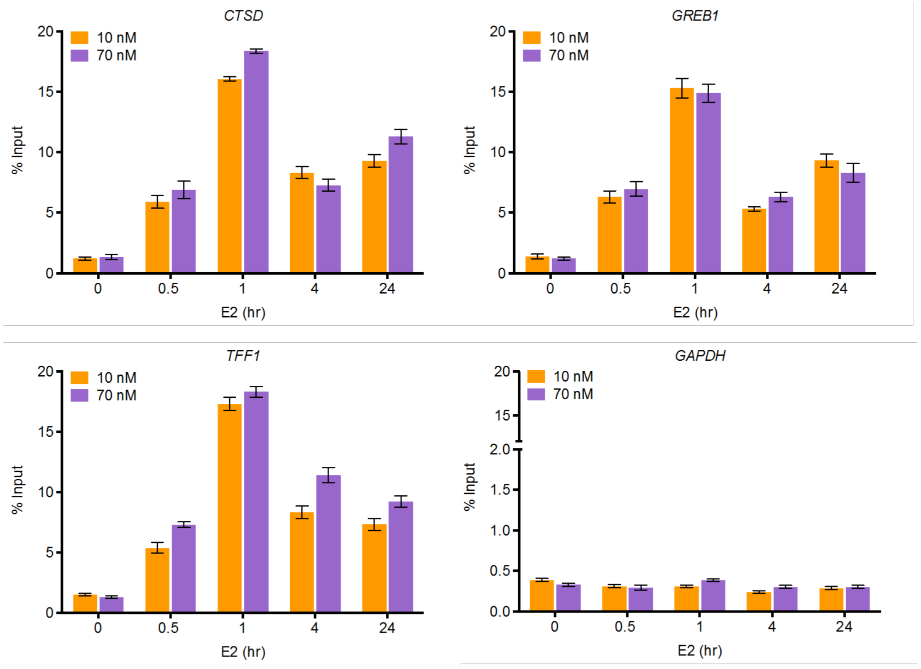

ERα binding of three estrogen-responsive genes (*CTSD*, *GREB1* and *TFF1*) and one gene not responsive to estrogen (*GAPDH*) by ChIP-qPCR.

Figure S4

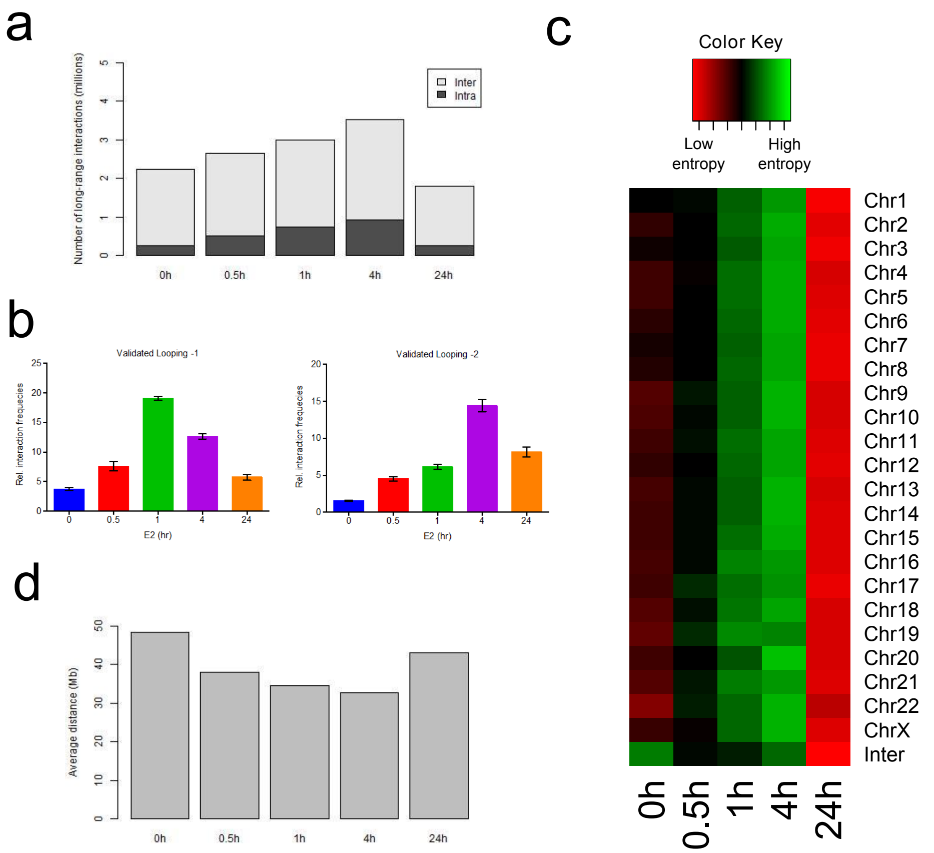

Statistical summary of intra- and interchromosomal interaction distributions over time. a) Number of intra- and interchromosomal interactions (Hi-C). b) Relative interaction frequencies measured by 3C-qPCR for two randomly choosen pairs of regions [chr9:67914751-67915000;chr4:190435001-190435250] and [chr1:121186751-121187000;chr19:32424751-32425000]. c) Entropy heatmap of interaction distributions (Hi-C). For a better visualization, row values have been scaled (Z-score). d) Average distance between interacting regions (Hi-C).

Figure S5

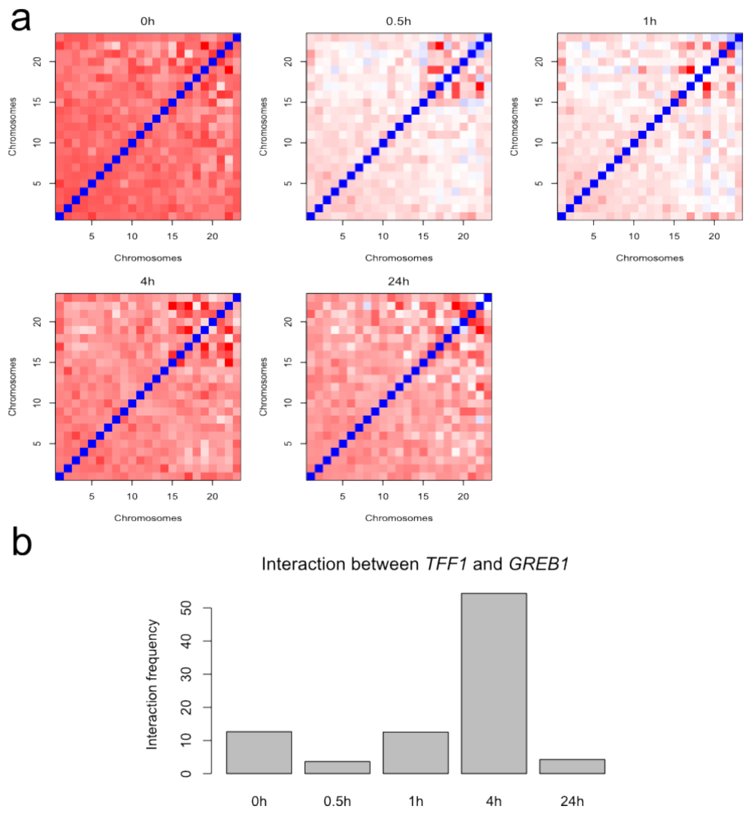

Effect of E2 on chromosome territories. a) Interchromosomal interaction frequencies between pairs of chromosomes. b) Interaction frequency between *FTT1* and *GREB1* loci (10 Mb window).

Figure S6

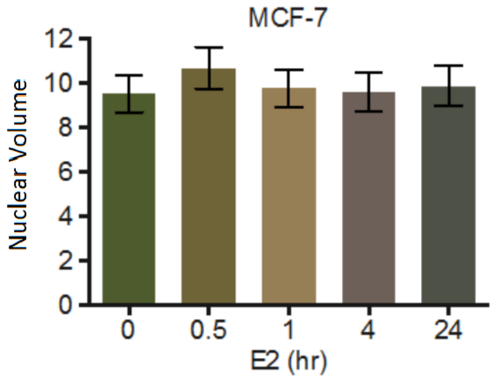

Effect of E2 on nuclear volume.

Figure S7

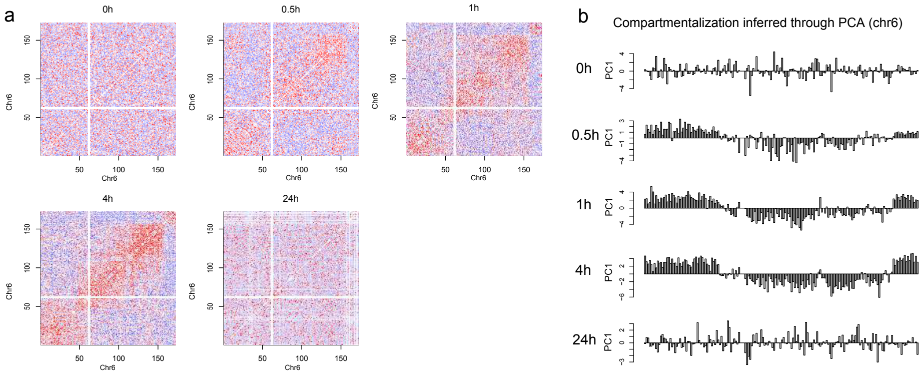

The effect of E2 on intrachromosomal interactions and chromatin compartmentalization, chromosome 6. a) Correlation heatmaps with 1 Mb binning. Red indicates positive correlation, and blue indicates negative correlation. b) First principal component (PC1) showing chromatin compartmentalization.

Figure S8

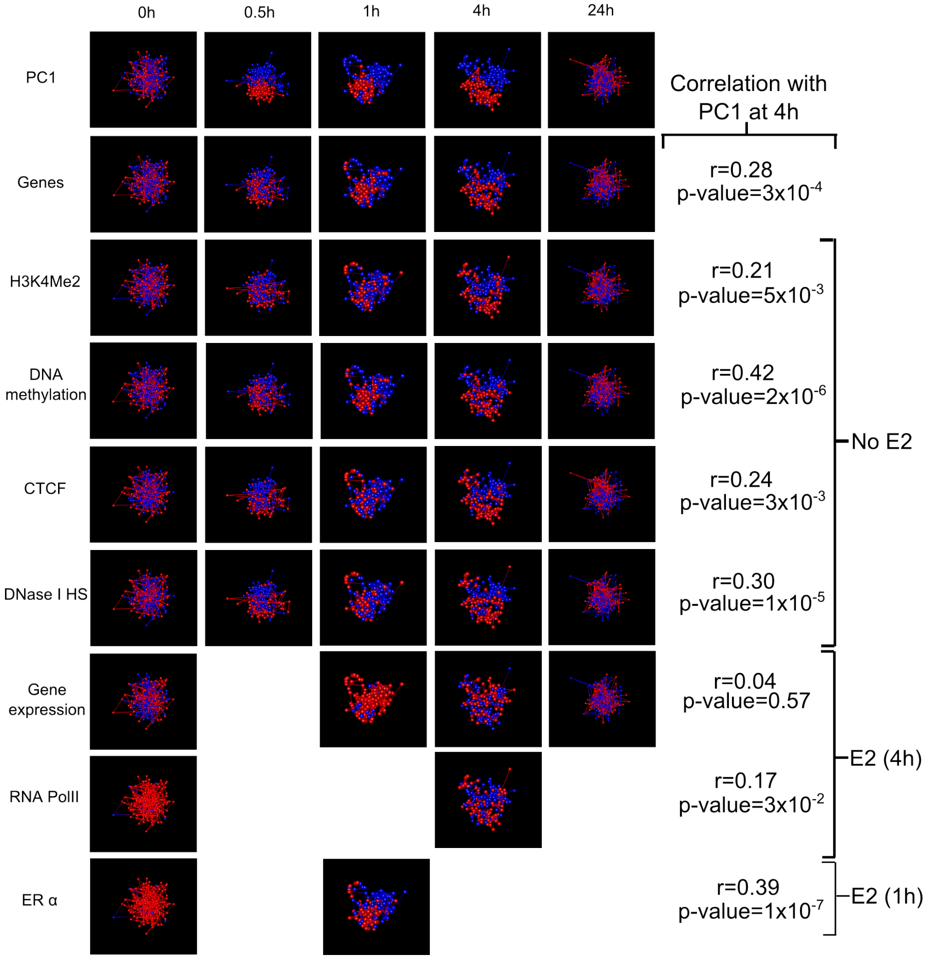

Influence of E2 on the 3D folding of genetic and epigenetic regions. Blue beads denote low values, while red beads represent high values of a genetic or epigenetic mark.

Figure S9

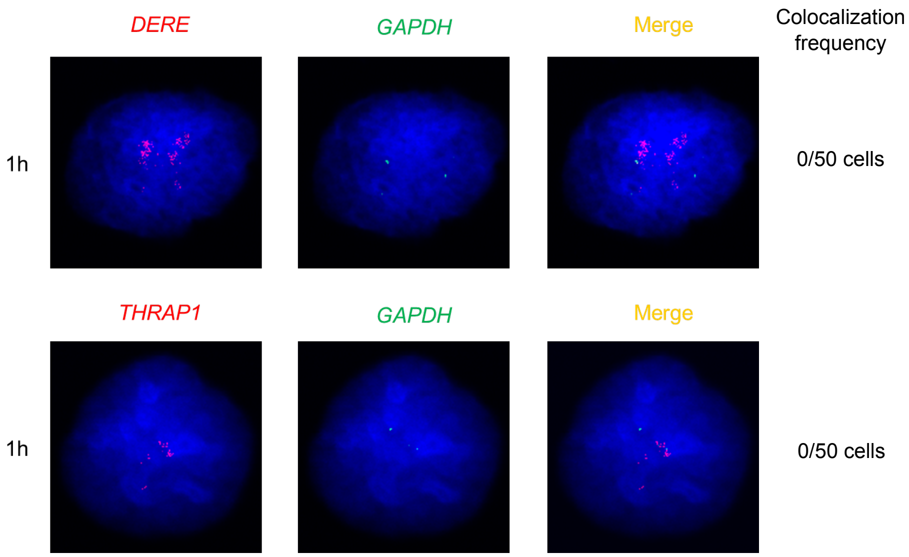

Interphase FISH control analysis between *GAPDH* [chr12:6643585-6647537] and *THRAP1* [chr17:60019966-60142643], and between *GAPDH* and 20q13 DERE [chr20:54155758-54155858].

Table S1

a

Lane	Replicate	Condition	Reads sequenced	Read mapped	InitialFilter	FilterRsiteStart	FilterDuplicate	FilterLarge	FilterExtreme
100707_s_1	1	0h	23714891	15762960	3787236	3411555	3274252	3215077	2963557
100729_s_1	1	0h	32654662	16669446	4445150	3997889	3860393	3799111	3524919
100729_s_2	2	0h	34728746	22619722	5261271	4775640	4496016	4399819	4002147
100729_s_3	2	0h	33616742	22464355	5234498	4748195	4472583	4377302	3983192
100707_s_2	1	0.5h	25832738	18442486	4306334	4007157	3844485	3760260	3419615
100729_s_4	1	0.5h	36096633	24016203	5554399	5177640	4913734	4798769	4351904
100729_s_5	2	0.5h	35788342	23519024	5477680	5099777	4851619	4750434	4335394
100729_s_6	2	0.5h	36113515	23681252	5441553	5068011	4816607	4706558	4271525
100707_s_3	1	1h	22301542	16188947	3888909	3556094	3314792	3245186	2961147
100707_s_4	1	1h	23303215	16848299	4129130	3772181	3515618	3445695	3150447
100729_s_7	2	1h	33267964	22449379	5394316	4942396	4523265	4429983	4038729
100729_s_8	2	1h	32849912	21738266	5180665	4741966	4349601	4258969	3883237
100707_s_5	1	4h	29436858	20768858	5724675	5254352	4642341	4560706	4183831
100707_s_6	1	4h	29022020	20483400	5615143	5157081	4546692	4464024	4085605
100804_s_1	2	4h	29982402	10175562	3783603	3419413	3249238	3215593	3026569
100804_s_2	2	4h	30485889	20118312	5987732	5476603	4904305	4830733	4461750
100707_s_7	1	24h	20719307	15118233	3362734	3113327	2867233	2806955	2582920
100707_s_8	1	24h	20556195	14807486	3266856	3020393	2802011	2750109	2541202
100804_s_3	2	24h	27231023	19191623	4257600	3945025	3603388	3528464	3245875
100804_s_4	2	24h	27092982	18824355	4177821	3865586	3547904	3476201	3206297

b

Replicate	Condition	Reads sequenced	Read mapped	InitialFilter	FilterRsiteStart	FilterDuplicate	FilterLarge	FilterExtreme
1	0h	56369553	32432406	8232386	7409444	7007798	6888607	6351475
2	0h	68345488	45084077	10495769	9523835	8755288	8567914	7769762
1	0.5h	61929371	42458689	9860733	9184797	8594602	8398510	7596512
2	0.5h	71901857	47200276	10919233	10167788	9494019	9285036	8432253
1	1h	45604757	33037246	8018039	7328275	6620633	6484090	5896429
2	1h	66117876	44187645	10574981	9684362	8617500	8436743	7661623
1	4h	58458878	41252258	11339818	10411433	8844431	8683315	7931010
2	4h	60468291	30293874	9771335	8896016	7927661	7821881	7250596
1	24h	41275502	29925719	6629590	6133720	5562987	5452187	5015015
2	24h	54324005	38015978	8435421	7810611	7035673	6890493	6331008

c

Condition	Reads sequenced	Read mapped	Initial Filtered	FilterRsiteStart	FilterDuplicate	FilterLarge	FilterExtreme
0h	124715041	77516483	18728155	16933279	15498412	15195377	13835097
0.5h	133831228	89658965	20779966	19352585	17816162	17417557	15759919
1h	111722633	77224891	18593020	17012637	14897915	14585422	13222156
4h	118927169	71546132	21111153	19307449	16361106	16097887	14763310
24h	95599507	67941697	15065011	13944331	12456223	12203341	11193041

Number of reads obtained after Hi-C data preprocessing. a) Lane level. b) Replicate level. c) Time point level.
